# Four-Year Outcomes of Left Main Percutaneous Coronary Intervention with a Bioresorbable Scaffold in the Circumflex Ostium

**DOI:** 10.1155/2022/7934868

**Published:** 2022-10-31

**Authors:** Andrejs Erglis, Inga Narbute, Dace Sondore, Sanda Jegere, Indulis Kumsars, Andis Dombrovskis, Karlis Grikis, Ieva Briede, Kristine Dombrovska, Karlis Trusinskis, Alona Grave, Martins Erglis, Martins Kalejs, Peteris Stradins, Uldis Strazdins

**Affiliations:** ^1^University of Latvia, Riga, Latvia; ^2^Pauls Stradins Clinical University Hospital, Riga, Latvia; ^3^Riga Stradins University, Riga, Latvia

## Abstract

**Objectives:**

The study aimed to investigate the long-term outcomes of a double stent scaffold strategy in patients with left main (LM) bifurcation lesions involving the ostium of the left circumflex artery (LCX), utilizing a drug-eluting stent (DES) in the LM extending into the left anterior descending artery (LAD) and a bioresorbable vascular scaffold (BVS) in the LCX ostium.

**Background:**

The high occurrence of in-stent restenosis of the LCX ostium is the major limitation of percutaneous coronary intervention (PCI) for LM lesions with a two-stent strategy.

**Methods:**

This was a single-center, prospective, single-arm study of 46 consecutively enrolled patients with a stable coronary artery disease and significant unprotected LM distal bifurcation disease. Patients underwent imaging-guided PCI using DES in the LM-LAD and BVS in the LCX using a T-stent or mini-crush technique. The primary outcome at four years was the composite of death, myocardial infarction, stroke, and target lesion revascularization (TLR).

**Results:**

At four years, the primary outcome was identified in 9 patients (19.6%). All events were TLRs except one myocardial infarction due to BVS thrombosis. Seven of the eight TLRs were a result of side branch BVS restenosis. Univariate predictors of the 4-year outcome were higher LDL cholesterol and BVS size ≤2.5 mm. On multivariate analysis, LCX lesion preparation with a cutting balloon and post-procedure use of intravascular ultrasound for optimization were found to be independent protective factors of MACE.

**Conclusions:**

In selected patients with LM distal bifurcation disease, an imaging-guided double stent scaffold strategy with DES in the LM and BVS in the LCX ostium was technically successful in all patients and was reasonably safe and effective for four years.

## 1. Introduction

The popularity of percutaneous coronary intervention (PCI) for the treatment of unprotected left main (LM) lesions has increased in the past few years because of advances in stent technologies and adjunctive pharmacotherapies. However, PCI of complex bifurcation lesions is still challenging in the drug-eluting stent (DES) era, and results are worse compared with simple coronary lesions. Although the provisional stent (PS) strategy is generally considered the default strategy for bifurcation lesions; there are scenarios in which the 2-stent strategy is initially necessary to improve the patency of both the main branch and the side branch. Unfortunately, the high occurrence of in-stent restenosis (ISR) of the left circumflex artery (LCX) ostium is a major limitation of the 2-stent strategy for unprotected left main (ULM) lesion [1], with a recent study reporting an ISR rate of 25.4% with this strategy (majority in the LCX ostium), vs. a rate of 6.3% with a provisional stent [2]. Conversely, the 3-year outcomes of the DKCRUSH-V Trial demonstrated that target lesion failure (TLF) was higher in the PS group vs. the DK crush group (16.9% vs. 8.3% *p*=0.005) and mainly driven by increased target vessel myocardial infarction (5.8% vs. 1.7%; *p*=0.017) and target lesion revascularization (10.3% vs. 5.0%; *p*=0.029). The definite or probable stent thrombosis (ST) rate at three years was 4.1% in the PS group and 0.4% in the DK group (*p*=0.006). Notably, DK crush was associated with a significant reduction in both primary and secondary endpoints for patients with complex lesions or at high risk [3].

BVS has the unique ability to restore vascular physiology and anatomical integrity, such as native tortuosity and angulation, with only a temporary scaffold necessary to maintain the patency of the vessel after the intervention [4, 5]. Studies have shown complete resorption of scaffold struts at 5 years [6, 7]. Therefore, it may provide a novel way to treat unprotected left main distal bifurcation lesions that would benefit from a two-stent strategy at the time of intervention but leave nothing behind to preclude later surgical revascularization or noninvasive imaging.

In the present study, we sought to assess the safety and efficacy at four years post-procedure of an IVUS-guided and OCT-optimized two-stent technique (mini-crush or T-stent strategy) using an everolimus-eluting platinum chromium coronary stent with a bioresorbable polymer coating (Synergy, Boston Scientific, Marlborough, MA) in the LM to left anterior descending (LAD) artery and a bioresorbable vascular scaffold (ABSORB, Abbott Vascular, Redwood City, CA) in the LCX ostium for the treatment of distal ULMCA true bifurcation stenosis.

## 2. Methods

### 2.1. Study Population

This was a prospective single-arm, single-center study of 46 consecutively patients enrolled from November, 2012, to December, 2015. The study included patients with a stable coronary artery disease and angiographically significant unprotected LM/LAD disease in whom revascularization with a two-stent technique was preplanned. The study inclusion criteria included patients >18 years old with symptomatic unprotected LM disease, including angiographic evidence of >50% diameter stenosis of a de novo true LM bifurcation lesion (Medina 1, 1, 1 or 0, 1, 1). Clinical exclusion criteria were acute myocardial infarction or stroke, anemia (Hb < 9 g/dl), and suspected intolerance to 1 of the study drugs. Clinical and angiographic (IVUS and OCT recommended) follow-up was conducted at 1 and 4 years post-procedure. The study included three patients (6.5%) with high SYNTAX scores (>32) who refused surgery and had been judged to be suitable candidates for PCI. Patients that did not return to the clinic were contacted for telephone follow-up. The protocol was approved by the ethics committee (ethics committee for clinical research at development society of Pauls Stradins Clinical University Hospital), and written consent was obtained from all patients. The study was funded in part by the national research program “biomedicine for public health” (BIOMEDICINE) VPP 2014/VPP2014-2017.

### 2.2. Procedures

All enrolled patients were scheduled to have LM PCI with two stents. A maximum of 24 hours before the procedure, all patients received 100 mg of aspirin. A loading dose of 300 mg of clopidogrel or ticagrelor 180 mg was administered before the index procedure. After the intervention, all patients received 100 mg/day aspirin for life and clopidogrel (75 mg/day) or ticagrelor 90 mg × 2 for at least 12 months. Unless precluded due to anatomical or clinical considerations, the treatment strategy was as follows:

Lesion assessment was performed on the main and side branches by angiography in two opposite projections and with intravascular ultrasound (iMAP, Boston Scientific, Marlborough, MA).

Modification of the atherosclerotic plaque was then performed with a cutting balloon (CB). The CB intervention was performed with a balloon-to-vessel ratio of 1 : 1, according to IVUS media-to-media to the vessel at the lesion site, and covered the entire lesion length. Balloon inflations were performed 3 times with increasing pressure throughout the lesion [8]. Standard PCI techniques were used to deploy a metallic DES in the LM/LAD and a BVS in the LCX ostium. The choice of a T-stent or mini-crush technique was at the discretion of the operator. Postdilatation with a noncompliant balloon and a final kissing balloon dilatation was then performed, followed by final intracoronary imaging with OCT (Lightlab Imaging, Westford, MA) and 40 MHz-IVUS (Boston Scientific Corporation, Marlborough, MA).

Procedural success was defined as a final residual stenosis <30% with TIMI flow grade 3 in either the main branch or the side branch.

**Figure 1:**
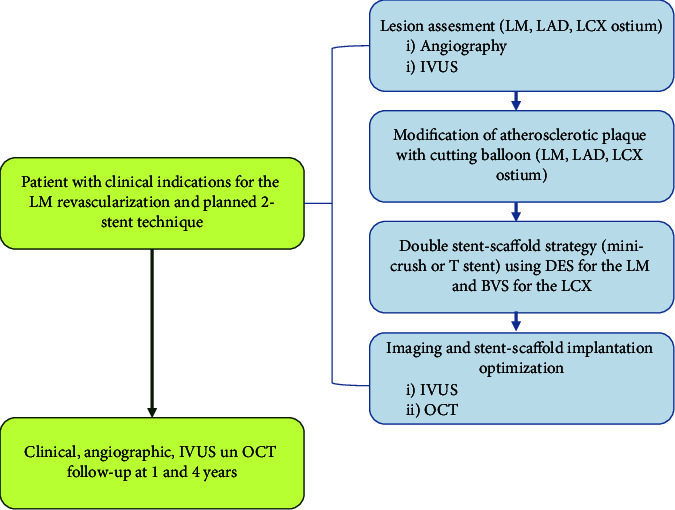
Study flowchart.

The study flowchart is presented in Figure 1.

### 2.3. Outcomes

The primary outcome measure was a composite of death, MI, stroke, and TLR, at 4 years. MI was diagnosed if the plasma level of the creatine kinase-myocardial band and/or troponin I/T increased to more than 3 times the upper limit of normal in no fewer than 2 blood samples. All deaths were considered cardiac in origin unless noncardiac reasons were indicated. TLR was defined as any repeat revascularization (percutaneous coronary intervention or CABG) for target lesions, respectively, in the presence of symptoms or objective signs of ischemia. ST was defined according to the Academic Research Consortium definition. All patients were scheduled for clinical and angiographic (recommended IVUS and OCT) follow-up at 1 year and 4 years. If the patient could not visit the hospital, clinical follow-up was obtained by telephone contact. In addition to the protocol-mandated follow-up, repeat coronary angiography was performed if clinically indicated due to symptoms and signs of myocardial ischemia.

### 2.4. Quantitative Coronary Analysis, IVUS, and OCT

All angiographic (pre, post, and follow-up) characteristics were analyzed offline by quantitative coronary angiography (QCA) using Cardiovascular Angiography Analysis System (CAAS) software 5.11.2, 2013 (Pie Medical Imaging, Maastricht, The Netherlands). QCA data included reference diameter and minimal luminal diameter at 6 lesion locations (ostial and body left main, proximal and mid part LAD, ostial and mid part LCX) where the landing of stent and scaffold was planned.

Preprocedural and postprocedural intravascular ultrasound (IVUS) and optical coherence tomography (OCT) at the index procedure and follow-up were used according to our center protocol to obtain more accurate information on the disease status and evaluate stent apposition after post-dilatation.

### 2.5. Statistical Analysis

All analyses were performed from the time of enrollment in the intention to treat population. Data were analyzed using SPSS version 21.0 (Statistical Package for the Social Sciences) software. Continuous variables are expressed as the mean ± SD. Categoric variables were tested using contingency table analyses (exact or Chi-square approximations), and continuous variables were tested using an unpaired student's *t*-test. Univariate logistic regression was used to identify the predictors of the primary outcome. Variables with probability values < 0.05 in individual analyses were included in the multivariate analysis. A *p* value of <0.05 was considered to be statistically significant.

## 3. Results

Patient baseline demographic and clinical characteristics are presented in Table 1. The mean age of patients was 66.1 ± 8.0 years, and the majority were males (73.9%). Dominant cardiovascular risk factors were arterial hypertension (95.7%) and dyslipidemia (87.0%). The history of myocardial infarction was present in 30.4% of patients; 45.7% prior had a percutaneous coronary intervention, 13.0% with non-insulin-dependent diabetes mellitus, family history of cardiovascular disease in 26.1%, and 63% of patients had chronic heart failure with a mean residual ejection fraction of 57.1 ± 9.2%. There are no data on statin therapy before the study, but the mean LDL at the time of the index procedure was 2.2 ± 0.8 mmol/l.

### 3.1. Procedural Characteristics and Quantitative Coronary Angiography

Procedural characteristics are presented in Table 2 and QCA data are presented in Table 3. Of the 46 procedures, 65.2% were performed through the femoral approach. Prior to predilation, IVUS of both branches was performed in 43 patients (93.5) %, CB predilation in LM-LAD in 84.7%. CB predilation of the LCX ostial in 82.6%. The mini-crush technique was employed in 69.6% of cases and the T-stent technique in 30.4% of cases. All patients were treated with BVS in the side branch, with a mean stent length of 15.83 mm and a diameter of 3.11 mm. “Final kissing” balloon dilation was performed in 91.3% of the cases. Post-procedure IVUS of both branches was performed in 40 patients (87.0%). The mean scaffold area in the LCX was 7.45 ± 1.57 mm^2^ by IVUS. All patients were treated successfully with 2 stents, DES in the LM and BVS in the LCX ostium, without major complications and were discharged in stable condition.

### 3.2. Primary Outcome Measures

The primary outcome measures a composite of death, stroke, myocardial infarction, and TLR, occurred in 9 (19.6%) patients during a 4-year follow-up. Nine events in seven patients (15.2%) occurred during the 1-year follow-up. Six events were clinically driven target lesion revascularization due to recurrent but stable angina symptoms with restenosis at the side branch, and there was one (2.2%) myocardial infarction resulting from stent thrombosis, which occurred 30 days after the index procedure. It was successfully treated by a PCI. An additional two TLRs (one in the LCX, one in the LM) occurred between one and four years of follow-up. At one year, angiographic follow-up was performed in all 46 patients (100%). At four years, angiographic follow-up was performed in 33 patients (71.7%) and by phone in 13 (28.3%). All patients (100%) received dual antithrombotic therapy for the recommended 12 months. 28 patients (60.9%) received clopidogrel and 18 patients (39.1%) received ticagrelor. Clinical outcomes at one and four years are presented in Table 4.

### 3.3. Predictors of Primary Outcome Measures

Using univariate logistic regression analysis, we identified parameters that predict primary outcome events. Results are presented in Table 5. Age, gender, and syntax score were not statistically significant predictors of events at 4 years, but LDL cholesterol was (OR 3.91; 95% CI 1.40–11.00). Procedural parameters that were significant predictors of an event were side branch plaque modification with cutting balloon, absorb scaffold diameter ≤2.5 mm in the side branch, and IVUS use post-procedure. In 9 patients (19.6%), the BVS diameter was ≤2.5 mm. The post-procedure IVUS was used more often in patients with BVS diameter >2.5 mm (*n* = 35, 94.6%) compared to patients with BVS diameter was ≤2.5 mm (*n* = 5, 55.6%), *p*=0.009. The stenting technique was not a significant predictor of MACE. The four-year MACE was observed in 5 patients with (15.6%) mini-crush and 4 patients (28.6%) with the T-stent technique (OR 0.46; 95% CI 0.10–2.08). On multivariate analysis, only LCX lesion preparation with cutting balloons and post-procedure use of intravascular ultrasound for optimization were found to be independent protective factors of MACE.

## 4. Discussion

To our knowledge, this is the first reported series on the treatment of true left main coronary artery bifurcation with a novel hybrid stent/scaffold strategy using a DES in the main branch and a BVS in the side branch. Considering the complexity of the patient group, this hybrid 2 stent/scaffold strategy demonstrated acceptable efficacy and safety with a rate of major adverse events similar to that reported in other studies of provisional and dual stent treatment strategies [3, 9–13].

In our study, 4-year mortality after PCI with a stent/scaffold hybrid approach for true left main bifurcation lesions was 0.0%. The NOBLE and EXCEL studies showed a higher mortality rate at 5 years in both the PCI (NOBLE 9%, EXCEL 13.0%) and CABG groups (NOBLE 9%, EXCEL 9.9%) [12, 13]. In the EXCEL study, the left main stenosis involved bifurcation in 81% of patients. A planned two-stent strategy was used in 35.0% of these patients. The death rate at 3 years was reported to be 11.0% [14]. The DKCRUSH-V trial was a randomized comparison of the double kissing (DK) crush planned 2-stent technique and provisional 1-stent technique in distal left main bifurcation lesions [15]. In the DK crush stenting group, the mortality rate at 3 years was 6.7% [3]. In a recently published European bifurcation club left main coronary stent study (EBC MAIN), in a randomized comparison of stepwise provisional vs. systematic double stenting strategies, the death rate in the dual stent group at 1 year was 4.2% [16]. The lower mortality in our study could be explained by the use of intracoronary imaging-guided pre-dilatation, stent sizing, and post-dilatation, or so-called iPSP strategy, which have been associated with a lower risk of cardiac events, including cardiac death, among patients undergoing PCI in complex coronary lesions [17]. In our study, preintervention and post-intervention imaging were used in more than 90% of our patients, while in EBC, main and DKCRUSH-V intravascular assessment was done in approximately 40% of the cases [15, 16]. Before stent implantation, plaque modification of the side branch and the main branch was performed in about 80% of the cases. Among patients randomized to a planned two-stent strategy in the EBC Main, plaque pretreatment with cutting balloon, rotablation, or lithotripsy was undertaken in 22% of main vessels and 15% of side branches. The lower mortality rate in the present study may be in part related to our learning curve gained from the first randomized trial (2004–2006) comparing DES and bare-metal stent for left main intervention, which showed a 2% death rate at 6 months and 7% at 3 years [8, 18].

In our study, 19.6% of patients underwent target lesion revascularization (TLR) 4 years after PCI. This finding is similar to the EXCEL study, where patients with planned two-stent strategies had 16.3% ischemia-driven revascularization of the left main complex at 3 years [14]. However, a lower TLR rate (5.0% at 3 years) was observed in the DK crush stenting group of the DKCRUSH-V trial [3]. Several factors could explain the differences in the TLR rate. Gaining experience with a new technique requires time and training. It should be noted that the majority of TLR cases (six in nine) occurred during the first 14 months of enrollment. Follow-up angiography could be associated with an increased incidence of TLR. It should be noted that 71.7% of our patients underwent angiographic follow-up at 4 years. In comparison, in the EXCEL study, only clinical follow-up was performed, but in the DKCRUSH-V trial, angiographic follow-up at 13 months and clinical follow-up at 3 years were performed. In the EBC MAIN study, the TLR rate at 1 year was 9.3% in the dual stent group [16]. It is lower than in our study (15.2% at 1 year), which can be explained by the EBC MAIN study requiring only a clinical follow-up, while our patients underwent angiographic follow-up at 1 year as well.

In our study, the rate of scaffold thrombosis was 2.2%, and no thrombosis occurred with the metal stent. In the two-stent subgroup of the EXCEL study, the rate of definite or probable stent thrombosis at 3 years was 2.8% [14]. In the DKCRUSH-V trial, the rate of definite or probable stent thrombosis at 3 years was 0.4% [3].

In our study, the rate of myocardial infarction was 2.2%. A higher rate of myocardial infarction (12.8%) was reported at 3 years in the two-stent subgroup of the EXCEL study [14].

The 0% mortality rate and one instance of stent thrombosis were found encouraging. Further, predictors of TLR, the major event rate driver, were associated primarily with inadequate lesion preparation, a BVS stent size of less than 2.5 mm, and a lack of intravascular imaging. These results reinforce our belief that adequate lesion preparation and comprehensive imaging are essential to good long-term outcomes with BVS in the ostial LCX.

Early experience with ABSORB BVS has generally demonstrated good procedural safety and angiographic success, as well as short to mid-term clinical outcomes and safety. However, the AIDA trial showed a higher definite and probable BVS thrombosis compared with DES, which was associated with more MI events but no significant difference in target vessel failure, death, or revascularization [14, 15]. In addition, the recently reported ABSORB II with a 3-year follow-up found significantly worse outcomes regarding device-oriented composite endpoints for Absorb BVS compared with Xience DES (10% versus 5%). Interestingly, neither angina status nor coronary functional vasomotion appeared to be superior in patients with Absorb BVS compared with metallic stents. These findings led to an FDA safety alert, followed by an announcement from the manufacturer regarding the discontinuation of normal sales [19]. The major concern of BVS is late and very late scaffold thrombosis [20]. However, in our study, only one early BVS stent thrombosis occurred (30 days after stent implantation), and no device thrombosis was observed through the 4-year follow-up. Data from the Absorb Japan trial [21] showed that, unlike standard DES, under and nonuniform device expansion was associated with greater negative remodeling and late lumen loss in ABSORB BVS, and this may in part account for the poorer outcomes after ABSORB BVS implantation compared with DES implantation in the lesions with suboptimal device expansion. Our series suggests that aggressive pre-dilatation and post-dilatation with the application of intracoronary imaging gives acceptable efficacy and safety using the BVS for complex lesions such as an unprotected left main bifurcation. Note that the one stent thrombosis occurred 40 days after the index procedure in a patient whose LCX lesion was not pretreated with a cutting balloon and the BVS diameter was 2.5 mm—both of which were predictors of the primary outcome.

The idea of “leaving nothing behind” after PCI is an intriguing concept for the treatment of bifurcation lesions. Our study suggests there may be a place for BVS in this lesion subset and further studies should be undertaken. [22].

### 4.1. Limitations

Limitations of the study include a small sample size, no control arm, and not all patients undergoing angiographic follow-up.

## 5. Conclusions

In conclusion, the use of a hybrid 2 stent/scaffold strategy with DES in the main branch and BVS in the side branch in selected patients with LM true bifurcation disease was technically possible and was reasonably safe and effective. The incidence of major events at short and long-term follow-up was similar to the published series of treatments with a two DES stent strategy. The number of patients and events in our cohort was small with no randomized control group, so firm conclusions cannot be made. Furthermore, larger scale and randomized studies are required.

## Figures and Tables

**Table 1:** Baseline patient characteristics.

Characteristics	All patients (*N* = 46)
Age, years	66.1 ± 8.9
Male gender	34 (73.9)
Hypertension	44 (95.7)
Dyslipidemia	40 (87.0)
Diabetes mellitus	6 (13.0)
Current smoking	13 (28.3)
Prior myocardial infarction	14 (30.4)
Prior percutaneous coronary intervention	21 (45.7)
Prior coronary artery bypass graft	0 (0)
Chronic heart failure	29 (63.0)
Peripheral artery disease	6 (13.0)
Family history of cardiovascular disease	12 (26.1)
Total cholesterol, mmol/l	4.0 ± 0.9
Low-density lipoprotein (LDL) cholesterol, mmol/l	2.2 ± 0.8
Left ventricular ejection fraction, %	57.1 ± 9.2
Syntax score	23.2 ± 5.3

Categorical variables are expressed as numbers and percentages. Continuous variables are indicated as mean ± SD.

**Table 2:** Procedural characteristics.

Characteristics	All patients (*N* = 46)
Transradial approach	16 (34.8)
7-F Guiding catheter	34 (73.9)
Pre-procedure IVUS of both branches	42 (91.3)
Cutting balloon pre-dilatation in LM-LAD	39 (84.8)
Cutting balloon diameter, mm	3.4 ± 0.3
Cutting balloon pre-dilatation in LCX	36 (78.3)
Cutting balloon diameter, mm	3.2 ± 0.3
*Stenting technique*	
T-stent	14 (30.4)
Mini-crush	32 (69.6)
*Stent LM-LAD*	
Synergy	42 (91.3)
Promus element	1 (2.2)
Promus premiere	1 (2.2)
Biofreedom	1 (2.2)
Xience V	1 (2.2)
LM/LAD stent length, mm	22.5 ± 7.3
LM/LAD stent diameter, mm	3.8 ± 0.3
BVS stent length, mm	15.8 ± 4.8
BVS stent diameter, mm	3.1 ± 0.4
Final kissing balloon dilatation	42 (91.3)
Post-procedure IVUS of both branches	40 (87.0)
Post-procedure OCT of both branches	42 (91.3)
Complications during angioplasty	5 (10.9)
Dissections covered with stent n	2 (4.3)
Dissections not covered with stent	1 (2.2)
Groin hematoma	2 (4.3)
Procedural success	46 (100)

Categorical variables are expressed as numbers and percentages. Continuous variables are indicated as mean ± SD.

**Table 3:** Quantitative coronary angiography data.

Parameter	Pre-procedure *N* = 46	Post-procedure *N* = 46	4-year follow-up *N* = 33
*Reference diameter, mm*			
Left main	3.54 ± 0.74	4.04 ± 0.37	4.05 ± 0.57
LAD	2.93 ± 0.58	3.60 ± 0.34	3.51 ± 0.42
LCX	2.82 ± 0.58	3.02 ± 0.38	2.88 ± 0.43

*Minimum lumen diameter, mm*			
Left main	2.18 ± 0.62	3.72 ± 0.33	3.62 ± 0.51
LCX	2.41 ± 0.63	3.47 ± 0.38	3.27 ± 0.43
LAD	1.69 ± 0.46	2.80 ± 0.37	2.29 ± 0.80

*% diameter stenosis, %*			
Left main	51.44 ± 16.07	8.37 ± 4.23	11.96 ± 5.99
LCX	50.93 ± 14.96	13.06 ± 5.18	15.84 ± 11.69
LAD	68.00 ± 13.59	8.09 ± 4.31	14.27 ± 6.12

Categorical variables are expressed as numbers and percentages. Continuous variables are indicated as mean ± SD.

**Table 4:** Clinical outcomes.

Cumulative events at 4 years	*N* = 46
Primary composite (death, myocardial infarction, stroke, TLR)	9 (19.6)
Death	0 (0.0)

Cardiovascular death	0 (0.0)
Myocardial infarction	1 (2.2)
Stroke	0 (0.0)
Clinically driven target lesion revascularization	9 (19.6)
LM-LAD DES restenosis	1 (2.2)
LCX BVS restenosis	7 (15.2)
LCX BVS thrombosis	1 (2.2)
Stent thrombosis	1 (2.2)

Cumulative events at 1 year	*N* = 46
Death	0 (0)
Cardiovascular death	0 (0)
Myocardial infarction	1 (2.2)
Stroke	0 (0)
Clinically driven target lesion revascularization	7 (15.2)
LM-LAD DES restenosis	0 (0)
LCX BVS restenosis	6 (13.1)
LCX BVS thrombosis	1 (2.2)
Stent thrombosis	1 (2.2)
Primary composite (death, myocardial infarction, stroke, TLR)	7 (15.2)

Categorical variables are expressed as numbers and percentages.

**Table 5:** Predictors of primary outcome measure by binary logistic regression.

Variable	*Univariate*		*Multivariate*	
	*p* value	OR (95% CI)	*p* value	OR (95% CI)
Age, years	0.615	1.02 (0.94–1.11)		
Male gender	0.769	1.30 (0.23–7.32)		
Dyslipidemia	0.372	0.42 (0.06–2.79)		
Diabetes mellitus	0.848	0.80 (0.08–7.84)		
Current smoker	0.238	2.49 (0.55–11.31)		
Heart failure	0.317	2.39 (0.43–13.10)		
Total cholesterol	0.021	2.84 (1.17–6.90)	0.226	
LDL	0.009	3.92 (1.40–11.00)	0.120	
Syntax score	0.671	0.97 (0.84–1.12)		
Cutting balloon diameter MB	0.868	0.80 (0.06–10.89)		
Cutting balloon diameter SB	0.460	3.58 (0.12–105.10)		
Cutting balloon side branch pre-dilatation	0.015	0.13 (0.02–0.63)	0.043	0.16 (0.03–0.94)
Stenting technique: mini-crush vs. T-stent	0.315	0.46 (0.10–2.08)		
LM-LAD stent length	0.068	1.10 (0.99–1.22)		
LM-LAD stent diameter	0.734	1.59 (0.11–23.48)		
Absorb length	0.611	1.04 (0.90–1.20)		
Absorb diameter	0.153	0.24 (0.03–1.71)		
Absorb size ≤2.5 mm	0.048	5.12 (1.02–25.81)	0.206	
Pre-intervention IVUS MB and SB	0.775	0.71 (0.06–7.71)		
Post-intervention IVUS MB and SB	0.008	0.07 (0.01–0.50)	0.026	0.09 (0.01–0.75)
Pre-intervention OCT MB and SB	0.775	1.42 (0.13–15.47)		

OR-odds ratio, MB-main branch, SB-side branch.

## Data Availability

All data that support the findings of this study are available from the corresponding author upon reasonable request.

## Abbreviations

DES: Drug-eluting stent
BVS: Bioresorbable vascular scaffold
LCX: Left circumflex artery
LM: Left main
LAD: Left anterior descending artery
TLR: Target vessel revascularization
PCI: Percutaneous coronary intervention
IVUS: Intravascular ultrasound
OCT: Optical coherence tomography.
